# Supplementary material for: Modeling glioblastoma heterogeneity as a dynamic network of cell states
Source: Mol Syst Biol. 2021 Sep 16;17(9):e10105. doi: 10.15252/msb.202010105 (PMC8444284; doi:10.15252/msb.202010105)
Supplement: Supplementary file 6 — Source Data for Figure 5 [file MSB-17-e10105-s004.zip › Figure5A_sourcedata/GSEA_3071/nosigdb_stateIII.GseaPreranked.1622795155562/FELDMAN_2020_M-EARLYG1.html]

Details for gene set FELDMAN\_2020\_M-EARLYG1[GSEA]

|  || Dataset | state33071 |
| Phenotype | NoPhenotypeAvailable |
| Upregulated in class | na\_neg |
| GeneSet | FELDMAN\_2020\_M-EARLYG1 |
| Enrichment Score (ES) | -0.61249363 |
| Normalized Enrichment Score (NES) | -3.369938 |
| Nominal p-value | 0.0 |
| FDR q-value | 0.0 |
| FWER p-Value | 0.0 |
Table: GSEA Results Summary

  

Fig 1: Enrichment plot: FELDMAN\_2020\_M-EARLYG1      
 Profile of the Running ES Score & Positions of GeneSet Members on the Rank Ordered List

  

| PROBE | GENE SYMBOL | GENE\_TITLE | RANK IN GENE LIST | RANK METRIC SCORE | RUNNING ES | CORE ENRICHMENT || 1 | MT2A |  |  | 31 | 0.499 | 0.0063 | No |
| 2 | COX8A |  |  | 63 | 0.448 | 0.0111 | No |
| 3 | FTL |  |  | 67 | 0.445 | 0.0231 | No |
| 4 | OST4 |  |  | 83 | 0.429 | 0.0316 | No |
| 5 | C12orf75 |  |  | 203 | 0.337 | 0.0105 | No |
| 6 | PTMA |  |  | 222 | 0.327 | 0.0152 | No |
| 7 | MZT2B |  |  | 244 | 0.313 | 0.0188 | No |
| 8 | LSM4 |  |  | 268 | 0.303 | 0.0215 | No |
| 9 | HNRNPA1 |  |  | 270 | 0.302 | 0.0299 | No |
| 10 | MYL9 |  |  | 293 | 0.293 | 0.0326 | No |
| 11 | STMN1 |  |  | 298 | 0.291 | 0.0399 | No |
| 12 | HMGN1 |  |  | 300 | 0.290 | 0.0480 | No |
| 13 | PSME2 |  |  | 310 | 0.289 | 0.0540 | No |
| 14 | HMGB1 |  |  | 318 | 0.286 | 0.0604 | No |
| 15 | H2AFZ |  |  | 386 | 0.265 | 0.0506 | No |
| 16 | TAGLN |  |  | 407 | 0.257 | 0.0529 | No |
| 17 | PHLDA1 |  |  | 589 | -0.259 | 0.0135 | No |
| 18 | GGH |  |  | 1439 | -0.302 | -0.1970 | No |
| 19 | UBE2C |  |  | 1942 | -0.332 | -0.3171 | No |
| 20 | GLIPR1 |  |  | 2390 | -0.364 | -0.4221 | No |
| 21 | TTYH1 |  |  | 2397 | -0.365 | -0.4132 | No |
| 22 | PRCP |  |  | 2489 | -0.372 | -0.4260 | No |
| 23 | CCNA2 |  |  | 2635 | -0.385 | -0.4524 | No |
| 24 | ACTA2 |  |  | 2721 | -0.393 | -0.4631 | No |
| 25 | PSAP |  |  | 2808 | -0.400 | -0.4738 | No |
| 26 | PCSK1N |  |  | 3346 | -0.470 | -0.5990 | Yes |
| 27 | MEST |  |  | 3363 | -0.473 | -0.5896 | Yes |
| 28 | PLAU |  |  | 3435 | -0.488 | -0.5939 | Yes |
| 29 | SERPINE1 |  |  | 3474 | -0.499 | -0.5894 | Yes |
| 30 | C16orf45 |  |  | 3489 | -0.504 | -0.5786 | Yes |
| 31 | DKK1 |  |  | 3556 | -0.530 | -0.5804 | Yes |
| 32 | TM7SF2 |  |  | 3558 | -0.530 | -0.5655 | Yes |
| 33 | CKB |  |  | 3572 | -0.536 | -0.5535 | Yes |
| 34 | IGFBP7 |  |  | 3611 | -0.554 | -0.5474 | Yes |
| 35 | FGFBP3 |  |  | 3649 | -0.578 | -0.5404 | Yes |
| 36 | TOP2A |  |  | 3671 | -0.594 | -0.5288 | Yes |
| 37 | HSP90B1 |  |  | 3757 | -0.681 | -0.5312 | Yes |
| 38 | ARL6IP1 |  |  | 3759 | -0.685 | -0.5119 | Yes |
| 39 | LIMA1 |  |  | 3774 | -0.706 | -0.4952 | Yes |
| 40 | SPARC |  |  | 3811 | -0.759 | -0.4828 | Yes |
| 41 | BEX1 |  |  | 3825 | -0.779 | -0.4638 | Yes |
| 42 | MOXD1 |  |  | 3837 | -0.804 | -0.4436 | Yes |
| 43 | CENPF |  |  | 3842 | -0.820 | -0.4211 | Yes |
| 44 | CLU |  |  | 3846 | -0.827 | -0.3982 | Yes |
| 45 | S100B |  |  | 3853 | -0.845 | -0.3755 | Yes |
| 46 | FABP7 |  |  | 3867 | -0.888 | -0.3534 | Yes |
| 47 | SCRG1 |  |  | 3869 | -0.904 | -0.3277 | Yes |
| 48 | TNC |  |  | 3874 | -0.951 | -0.3015 | Yes |
| 49 | PTN |  |  | 3884 | -1.022 | -0.2745 | Yes |
| 50 | CYR61 |  |  | 3887 | -1.037 | -0.2453 | Yes |
| 51 | GPM6B |  |  | 3889 | -1.047 | -0.2156 | Yes |
| 52 | ASPM |  |  | 3899 | -1.166 | -0.1845 | Yes |
| 53 | CTGF |  |  | 3901 | -1.185 | -0.1508 | Yes |
| 54 | IGFBP3 |  |  | 3903 | -1.199 | -0.1167 | Yes |
| 55 | PTPRZ1 |  |  | 3913 | -1.342 | -0.0805 | Yes |
| 56 | HIST1H4C |  |  | 3914 | -1.352 | -0.0418 | Yes |
| 57 | MALAT1 |  |  | 3924 | -1.583 | 0.0013 | Yes |
Table: GSEA details [plain text format]

  

Fig 2: FELDMAN\_2020\_M-EARLYG1: Random ES distribution      
 Gene set null distribution of ES for **FELDMAN\_2020\_M-EARLYG1**
